# Left Ventricular Isoperimetric Properties in Hypertrophic Cardiomyopathy: A CMR-Based Analysis of Ventricular Geometry

**DOI:** 10.3390/diagnostics16142142

**Published:** 2026-07-08

**Authors:** Maja Milošević Nale, Bojan Božić, Ivan Soldatović, Ljiljana Ranković-Ničić, Goran Lončar, Milica Milošević, Nikola Mitović, Vladimir Mihajlović, Ivana Petrović, Milovan Bojić, Milan Dobrić

**Affiliations:** 1 Institute for Cardiovascular Diseases “Dedinje”, Heroja Milana Tepića 1, 11000 Belgrade, Serbia; maja.a.milosevic@gmail.com (M.M.N.); ljiljanabg80@yahoo.com (L.R.-N.); loncar_goran@yahoo.com (G.L.); milosevic.a.milica13@gmail.com (M.M.); vlada_sd@yahoo.com (V.M.); petrovicivana4@gmail.com (I.P.); dedinje@ikvbd.com (M.B.); 2 Institute of Physiology and Biochemistry “Ivan Djaja”, Faculty of Biology, University of Belgrade, Studentski trg 16, 11000 Belgrade, Serbia; 3 Faculty of Medicine, University of Belgrade, Dr Subotića 8, 11000 Belgrade, Serbia; ivan.soldatovic@med.bg.ac.rs (I.S.); nikolamitovic@gmail.com (N.M.); 4 Faculty of Medicine, University of Banja Luka, Save Mrkalja 14, 78000 Banja Luka, Bosnia and Herzegovina

**Keywords:** hypertrophic cardiomyopathy, cardiac magnetic resonance, left ventricular geometry, ventricular remodeling, isoperimetric index, fractal analysis

## Abstract

**Background/Objectives:** Hypertrophic cardiomyopathy (HCM) is a complex myocardial disease in which structural remodeling extends beyond conventional parameters such as maximal wall thickness (MWT). This study aimed to evaluate isoperimetric analysis as a geometry-based approach for characterizing left ventricular (LV) morphology and to compare its performance with fractal analysis in distinguishing HCM from non-HCM subjects. **Methods:** This retrospective study included 120 subjects (60 HCM and 60 controls) who underwent clinically indicated CMR. Endocardial contours were delineated on short-axis cine images at end-diastole. The isoperimetric index (IPI), cavity-corrected IPI (CC-IPI), and fractal dimension (FD) were calculated. Group comparisons, correlations, and receiver operating characteristic analyses were performed. **Results:** Patients with HCM showed significantly higher IPI and CC-IPI values than controls (*p* < 0.001), including in sex-stratified analyses. IPI and CC-IPI correlated strongly with MWT (r = 0.867) and LV mass index (r = 0.807 and 0.803, respectively), moderately with native T1 (r = 0.567), and weakly with end-diastolic volume index (r = 0.256 and 0.253, respectively) and stroke volume (r = 0.237 and 0.229, respectively). No association was observed with late gadolinium enhancement. IPI and CC-IPI showed a strong linear correlation (R^2^ = 0.997), with both indices demonstrating high discriminatory performance (AUC = 1.00). **Conclusions:** HCM is associated with notable alterations in LV endocardial geometry beyond myocardial hypertrophy alone. IPI and CC-IPI provide simple, size-normalized, and physiologically interpretable morphologic descriptors that complement conventional CMR parameters and FD-based contour complexity analysis.

## 1. Introduction

Hypertrophic cardiomyopathy (HCM) is a common primary cardiomyopathy (CMP), defined by a left ventricular (LV) wall thickness ≥ 15 mm in any myocardial segment that could not be explained solely by loading conditions [1]. The prevalence of HCM in the general population is estimated to be approximately 1:500 based on clinical expression of the disease phenotype [2], while some imaging and genotype studies suggest even greater HCM prevalence, reaching up to 1:200 of the population [1].

HCM is morphologically characterized by primary hypertrophy of the myocardium, myocyte disarray and myocardial fibrosis [3]. On the macroscopic level, the key diagnostic feature of HCM is increased LV maximum wall thickness (MWT), although the classical imaging definition of hypertrophy relies on increased LV mass [4]. Primary mitral valve and sub-valvular apparatus abnormalities, including papillary muscles (PMs), are common in patients with HCM and can contribute to developing systolic anterior motion (SAM) of the mitral valve and dynamic LV outflow tract obstruction (LVOTO) [1,4,5].

Cardiac magnetic resonance (CMR) remains the gold standard diagnostic method for patients with HCM, by providing differentiation of HCM from other entities characterized by LV hypertrophy, characterization of the HCM phenotype, and quantification of ventricular volumes and function, as well as tissue characterization [1,3,4,6,7,8,9]. Furthermore, CMR-derived parameters such as MWT, the extent of late gadolinium enhancement (LGE), left ventricular ejection fraction (LVEF), the presence of an LVOTO and an LV apical aneurysm are integrated into current calculators for the assessment of sudden cardiac death risk or considered important risk modifiers [1,8,9].

The 2025 European Association of Cardiovascular Imaging (EACVI) Clinical Consensus Statement emphasizes limitations in current diagnostic definitions of HCM that rely predominantly on MWT and suggests that more sophisticated, often multi-modal assessments may be needed [4]. Although MWT remains central to diagnosis, it represents a simplified descriptor that does not fully reflect the spatial distribution of hypertrophy, the dynamic relationship between cavity and myocardium, nor the global three-dimensional ventricular configuration [1,4]. One promising approach involves geometric characterization of the LV, as ventricular shape and structural organization are closely linked to cardiac biomechanics, including wall stress distribution, mechanical efficiency, and remodeling patterns [10]. Among the proposed approaches, particular interest has been directed toward the analysis of endocardial trabeculation [11,12,13,14,15]. Recent studies have demonstrated that a substantial proportion of LV myocardium in patients with HCM may be trabeculated, in some reports accounting for approximately 25–30% of total myocardial mass [11]. Increased myocardium trabeculation has been associated with adverse structural remodeling, systolic impairment, greater fibrosis burden, and worse clinical outcomes [11,12]. Importantly, standard contouring-based CMR volumetric approaches may not explicitly quantify the geometric complexity introduced by PMs and trabecular structures, potentially leading to underestimation of LV structural heterogeneity [5,11,12].

To overcome these limitations, quantitative descriptors of ventricular geometry have been proposed, with fractal dimension (FD) being one of the most widely investigated metrics [13,14,16,17]. Clinically, FD has been interpreted as a quantitative measure of trabecular network complexity with potential implications for phenotypic characterization and risk stratification [13,14,17]. However, FD is inherently contour-based and does not account for effective cavity area or volume, thereby reflecting surface complexity rather than global ventricular geometry [16]. Furthermore, in HCM, where PMs are often hypertrophied or displaced, their exclusion may further limit geometric characterization [5]. Additionally, fractal measurements are sensitive to image acquisition and spatial resolution, which may affect reproducibility [13,16]. Thus, despite advances in quantifying local morphological complexity, an integrative metric that extends beyond contour complexity and captures global ventricular morphology remains lacking.

Considering these limitations, there is a need for a novel parameter that integrates the information on endocardial border complexity and effective cavity area or volume. In this context, the isoperimetric principle provides a geometry-based quantitative approach to the assessment of LV structure. In patients with HCM, in whom asymmetric remodeling and architectural distortion alter LV geometry, an isoperimetric approach may provide a more integrative and clinically interpretable descriptor of ventricular structure. Accordingly, the present study investigates an isoperimetric-based geometric model as a novel descriptor of LV architecture in HCM patients.

## 2. Materials and Methods

### 2.1. Study Population and Ethical Considerations

This was a retrospective, single-center, observational study including patients whose previously acquired CMR studies were identified through the institutional Picture Archiving and Communication System (PACS) database. All CMR examinations were performed as part of routine clinical care and not for research purposes. The study included 120 adult individuals (>18 years) evaluated at the Institute for Cardiovascular Diseases “Dedinje”, Belgrade, Serbia, between December 2022 and February 2026, who were divided into two groups. The HCM group comprised 60 patients with either a confirmed genetic diagnosis or a strong clinical suspicion of HCM in accordance with current guidelines [1], defined as LV MWT ≥ 15 mm in any myocardial segment not explained solely by loading conditions. Exclusion criteria for the HCM group included substantial LV remodeling consistent with the end-stage phase of HCM or a history of septal reduction therapy (surgical myectomy or alcohol septal ablation). The control group consisted of 60 individuals referred for clinically indicated CMR examinations due to arrhythmias, suspected prior acute coronary syndrome, suspected myocarditis, or other findings requiring exclusion of structural heart disease. Following comprehensive CMR evaluation, only subjects with normal left ventricular morphology and function and no evidence of cardiomyopathy or any other structural cardiac abnormality were included and classified as HCM-negative controls. In both groups, CMR examinations with inadequate image quality due to significant artifacts were excluded from the analysis.

The study was conducted in accordance with the principles of the Declaration of Helsinki and was approved by the Ethics Committee of the Institute for Cardiovascular Diseases “Dedinje”, Belgrade, Serbia (protocol No. 6348). Given the retrospective design of the study, the use of previously recorded clinical data available in the institutional database, and the absence of any additional interventions involving the participants, the requirement for written informed consent was waived by the Ethics Committee. All patient data were de-identified prior to analysis.

### 2.2. Cardiovascular Magnetic Resonance

All participants underwent CMR imaging prior to inclusion in the study according to the standardized institutional protocol of the “Dedinje” Institute, based on current international CMR guidelines [18,19]. Examinations were performed using a 1.5-T scanner (Signa Artist, General Electric Healthcare, Milwaukee, WI, USA). The imaging protocol included standard clinical acquisitions comprising localizers, three long-axis views (2-, 3-, and 4-chamber), a complete short-axis (SAX) cine stack covering the entire LV from base to apex, and black-blood (BB) sequences. Cine images were acquired using balanced steady-state free precession sequences during breath-hold with retrospective electrocardiographic gating.

Data acquisition and reporting were performed in accordance with contemporary CMR recommendations [20] and included assessment of ventricular morphology, function, and tissue characteristics. Functional evaluation comprised quantification of left and right ventricular EF, LV stroke volume (SV), LV indexed end-diastolic and end-systolic volumes (EDVi and ESVi) and global and regional wall motion. Structural assessment included measurement of MWT, chamber volumes, and myocardial mass. Detailed morphological characterization focused on the HCM phenotype, SAM of the mitral valve, LVOTO, apical aneurysm, and papillary muscle abnormalities. Tissue characterization included the assessment of myocardial edema using T2 mapping and BB sequences, as well as the evaluation of focal and diffuse myocardial fibrosis using LGE and native T1 mapping.

### 2.3. Standard CMR Image Analysis

Quantitative analysis was performed on SAX cine images derived from balanced steady-state free precession sequences. Standard morphological and functional parameters, including LV EDV and ESV, SV, EF, myocardial mass, and MWT, were assessed using commercially available software (cvi42 6.4.2, Circle Cardiovascular Imaging Inc., Calgary, AB, Canada). Endocardial and epicardial contours were semi-automatically delineated with manual correction at end-diastole and end-systole, with PM and trabeculations excluded from the myocardial mass. Volumes and myocardial mass were indexed to body surface area. Regional wall motion and hypertrophy distribution were evaluated using the 17-segment model.

LGE images were analyzed for the presence, pattern, and extent of myocardial fibrosis. Native T1 and T2 mapping were performed using standardized acquisition protocols, and global values were derived from mid-ventricular SAX slices by placing regions of interest within the mid-ventricular septal myocardium, carefully avoiding blood pool contamination. LVOTO was assessed using cine imaging based on the presence of flow acceleration and signal void consistent with turbulent flow.

### 2.4. Isoperimetric and Fractal Analysis

For each participant, manual delineation of the LV endocardial contour was performed on selected SAX cine slices from base to apex. The geometric analysis also included trabeculations, PMs, and subvalvular mitral structures, ensuring comprehensive representation of endocardial morphology. Manual segmentation was performed by the primary investigator and subsequently reviewed and validated by consensus of two ESC-accredited cardiologists with expertise in CMR, ensuring methodological consistency and clinical accuracy. Based on the extracted binary endocardial masks, fractal and isoperimetric analyses were performed using custom-written code in Python 3.13.3 (Python Software Foundation, Wilmington, DE, USA), implemented and executed in Visual Studio Code 1.119.0 (Microsoft Corporation, Redmond, WA, USA).

All CMR analyses were performed on anonymized datasets. However, due to the characteristic morphological features of HCM on cine imaging, complete blinding to group allocation was not feasible.

Fractal dimension was calculated using the box-counting method. The binarized endocardial contour was overlaid with grids of progressively decreasing box size (ε), and the number of occupied boxes N(ε) was determined. FD was obtained as the slope of the linear regression in the log–log plot of log N(ε) versus log(1/ε).

Isoperimetric analysis was performed using length/area (L/A) relationships derived from the classical geometric framework. Because L scales linearly with length, whereas A scales quadratically, direct normalization by area (L/A) would yield a dimensionally inconsistent measure dependent on ventricular size. Therefore, L was normalized by the square root of A (L/√A), resulting in a dimensionless and scale-invariant index suitable for comparison across ventricles (and slices) of different sizes. Isoperimetric analysis was performed in parallel using two complementary indices:

- Conventional isoperimetric index (IPI), defined as

IPI = L/√A

where L denotes the length of the LV endocardial contour, and A represents the total area enclosed by the endocardial contour.

- Cavity-corrected isoperimetric index (CC-IPI), defined as

CC-IPI = L/√(A -ΣAinner)

where L denotes the length of the LV endocardial contour, A represents the total enclosed endocardial area, and ΣAinner corresponds to the cumulative area of internal myocardial structures within the LV cavity.

While the classical IPI reflects boundary complexity relative to total enclosed area, the corrected index accounts for the effective cavity area by excluding internal myocardial structures, thereby providing a more physiologically representative measure of global ventricular geometry.

All calculations were performed on a per-slice basis (7–15 slices per subject, depending on LV size). Subject-level IPI, CC-IPI, and FD values were obtained as the arithmetic mean of measurements across all analyzed slices. In addition, the standard deviation (SD) and coefficient of variation (CV) were calculated to assess intra-ventricular variability of geometric parameters. Descriptive statistics and distribution of isoperimetric indices in HCM and control groups are presented in Supplementary Table S1.

### 2.5. Contour Extraction

**Figure 1:**
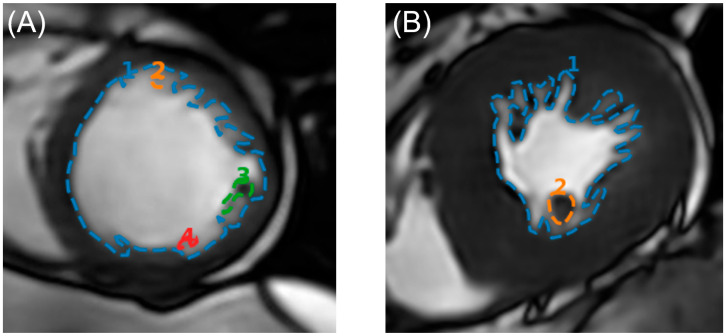
Endocardial contour tracing used for geometric analysis. Representative (mid-cavity) SAX cine CMR images demonstrating manual delineation of the LV endocardial contour in a Control subject (**A**) and a patient with HCM (**B**).

Representative examples of endocardial contour tracing are shown in Figure 1. The LV endocardial border was manually delineated on short-axis cine CMR images at end-diastole. Manual segmentation was selected because it enabled more accurate delineation of trabecular structures and irregular endocardial borders relevant to the geometric analyses performed in this study. The average manual segmentation time was approximately 5–10 min per patient, with variability related to the number of short-axis slices and the complexity of ventricular geometry. Contour tracing followed the inner myocardial boundary along the entire LV circumference. PMs, cross-sectioned trabeculations, and other intracavitary myocardial structures were delineated separately within the LV cavity and were not included in the endocardial contour. For geometric analysis, three parameters were extracted from the binary masks: the endocardial contour length (L), the total enclosed endocardial area (A), and the cumulative area of intracavitary myocardial structures (Ainner).

### 2.6. Statistical Analysis

Continuous variables are presented as mean ± standard deviation or median (interquartile range), as appropriate, while categorical variables are expressed as counts and percentages. Group comparisons were performed using Student’s *t*-test or χ^2^ test, as appropriate. Pearson correlation analysis was used to assess associations between continuous variables, and linear and logistic regression analyses were applied to evaluate relationships between the studied parameters. Diagnostic performance was assessed using receiver operating characteristic (ROC) analysis, with the area under the curve (AUC) used as a measure of discriminative ability. A *p*-value < 0.05 was considered statistically significant. Statistical analyses were performed using IBM SPSS Statistics 31.0 (IBM Corp., Armonk, NY, USA) and R 4.5.0 (R Foundation for Statistical Computing, Vienna, Austria).

## 3. Results

### 3.1. Study Population and Baseline Characteristics

Baseline characteristics of the study population are presented in Table 1. There was no significant difference in age between groups (42.95 ± 11.8 years in HCM group vs. 41.07 ± 11.15 years in the control group, *p* = 0.371), although male sex was more prevalent in the HCM group (73.3% vs. 55.0%, *p* = 0.036). LV EF was similar between groups (61.22 ± 6.06% in the HCM group vs. 60.18 ± 3.83% in the control group, *p* = 0.266), indicating preserved LV systolic function. In contrast, parameters reflecting myocardial hypertrophy and tissue remodeling, such as MWT, LV mass, and native T1, were significantly increased in the HCM group (all *p* < 0.001). In addition, EDV, EDVi and SV showed statistically significant differences (all *p* < 0.05).

### 3.2. Morphological and Phenotypic Characteristics of the HCM Group

The morphological and phenotypic characteristics of the HCM group are summarized in Table 2. Among HCM patients, SAM of the mitral valve was present in 45% and LVOTO in 35% of cases, while apical papillary muscle displacement was identified in 40% of patients. The most prevalent phenotypic subtype was asymmetric hypertrophy (88.3%), followed by concentric (8.3%) and apical forms (3.3%).

### 3.3. Gender-Based Differences in Isoperimetric Indices and CMR Parameters in the Control Group

IPI, CC-IPI and FD have been calculated for both groups. Interestingly, in the control group, both the IPI and the CC-IPI were significantly higher in males compared with females (Table 3). In contrast, FD values did not differ significantly between sexes (Table 3).

Gender-based differences were also observed across multiple conventional CMR parameters within the control group (Table 4). While age was comparable between males and females (*p* > 0.05), a small but statistically significant difference was observed in LVEF (*p* = 0.048). Males also demonstrated significantly higher values of MWT, EDV, EDVi, SV, and LV mass index (all *p* < 0.05). In addition, significant differences were noted in native T1 mapping values between sexes (*p* < 0.05).

### 3.4. Comparison of Isoperimetric Indices and Fractal Dimension Between HCM and Controls in the Overall Cohort and Stratified by Gender

**Figure 2:**
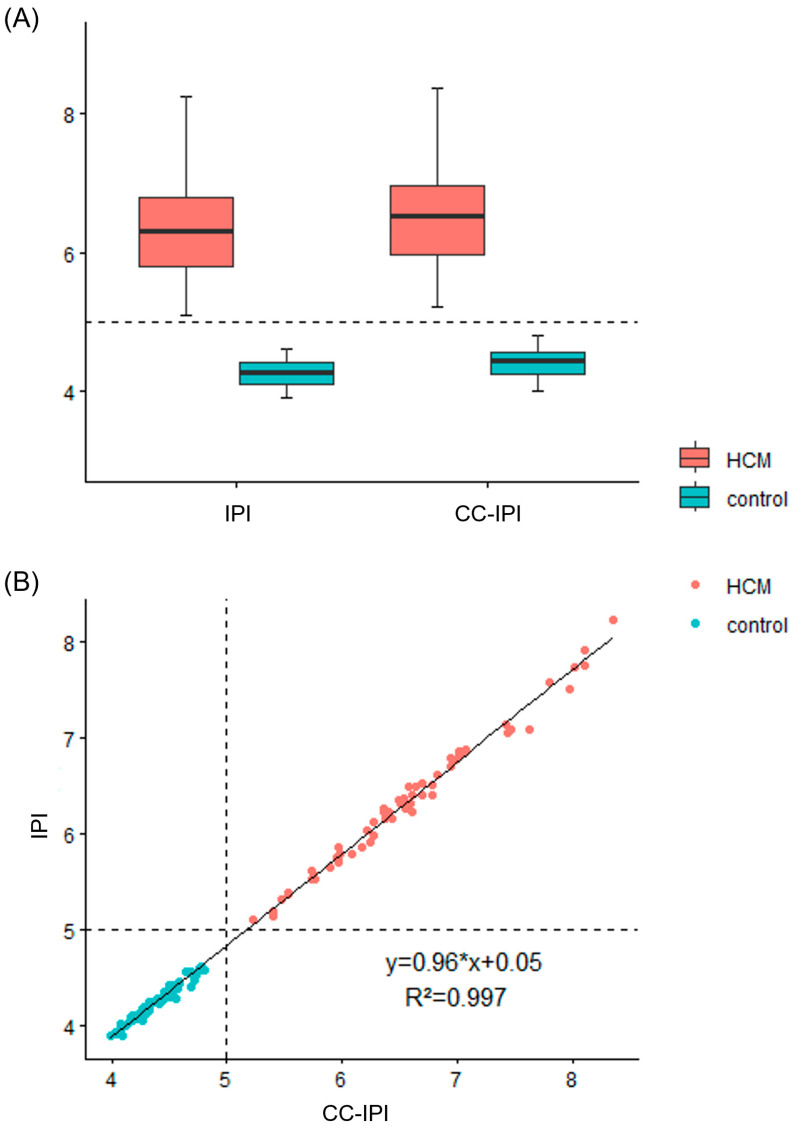
Relationship and group distribution of isoperimetric indices. (**A**) Box plot illustrating the distribution of IPI and CC-IPI values in HCM patients and controls; (**B**) scatter plot demonstrating the linear relationship between IPI and CC-IPI (R^2^ = 0.997). Abbreviations: HCM—hypertrophic cardiomyopathy; IPI—conventional isoperimetric index; CC-IPI—cavity-corrected isoperimetric index.

Both IPI and CC-IPI were significantly higher in HCM patients compared with controls in the overall cohort (Table 5 and Figure 2A, all *p* < 0.001). Similar findings were observed for FD (Table 5 and Figure S1, all *p* < 0.001). These differences remained highly significant after stratification by gender, with consistently elevated values observed in both male and female subgroups (all *p* < 0.001), indicating that the geometric alterations associated with HCM were independent of gender (Table 5). Linear regression analysis demonstrated a strong correlation between IPI and CC-IPI (R^2^ = 0.997, *p* < 0.001), reflecting a high level of agreement between the two formulations. The scatter plot showed a tightly clustered linear distribution across the entire range of values and clear separation between investigated groups (Figure 2B). Additionally, correlation analyses between FD and the isoperimetric indices (IPI and CC-IPI) were performed (Figures S2 and S3, respectively). Although strong positive correlations were observed (R^2^ = 0.9162 and 0.9144, respectively), they were found to be lower than the correlation between IPI and CC-IPI (R^2^ = 0.997).

### 3.5. Diagnostic Performance of Isoperimetric Indices and Fractal Dimension

The receiver operating characteristic curve analysis demonstrated full discriminatory performance of IPI for identifying HCM, with an AUC of 1.00 (95% CI 1.00–1.00, *p* < 0.001) (Table 6). The optimal cut-off value for IPI was 4.86, yielding a sensitivity of 100% and specificity of 100% (Youden index = 1.00). Comparable diagnostic performance was observed for CC-IPI, with an AUC of 1.00 (95% CI 1.00–1.00, *p* < 0.001) and an optimal cut-off value of 5.01, also achieving 100% sensitivity and 100% specificity (Youden index = 1.00). In addition, although FD also showed strong discriminatory ability between control and HCM subjects (Table 5), a small overlap between the two distributions was observed (AUC of 0.999 (95% CI 0.997–1.000) (Figure S1, Table 6). When the FD threshold was set to preserve full HCM sensitivity FD ≥ 1.0808, all HCM cases were correctly identified (60/60), corresponding to 100% sensitivity. However, two subjects from the control group were classified as HCM, resulting in a specificity of 96.67%. Thus, FD may be considered a supportive screening parameter, particularly useful for minimizing false-negative HCM classification, whereas IPI and CC-IPI provide complete cohort separation and were retained as the primary diagnostic indices.

### 3.6. Correlation Between Isoperimetric Indices with Conventional CMR Parameters

Significant associations were observed between isoperimetric indices and established structural CMR parameters (Table 7). Both IPI and CC-IPI demonstrated strong positive correlations with MWT (r = 0.867, *p* < 0.001 for both parameters) and indexed LV mass (r = 0.807, *p* < 0.001 and r = 0.803, *p* < 0.001, respectively). Moderate positive correlations were observed with native T1 values (r = 0.567, *p* < 0.001 for both parameters). Weak but statistically significant correlations for both IPI and CC-IPI were also observed with EDV (r = 0.223, *p* = 0.014 and r = 0.217, *p* = 0.017, respectively) and EDVi (r = 0.256, *p* = 0.05 and r = 0.253, *p* = 0.05, respectively). Additionally, significant correlations with SV were observed for both IPI (r = 0.237, *p* < 0.05) and CC-IPI (r = 0.229, *p* = 0.012). In contrast, no significant association was observed between isoperimetric indices and the extent of LGE (r = 0.210, *p* = 0.107 for IPI and r = 0.223, *p* = 0.087 for CC-IPI).

## 4. Discussion

The relationship between ventricular geometry and cardiac function has long been recognized. Early work by Tomlinson demonstrated that ventricular performance is closely related to geometric characteristics of the left ventricle, while Ganau et al. showed that ventricular remodeling reflects the interplay between cavity size and myocardial thickness rather than myocardial mass alone [21,22]. Consistent with Laplace’s law, alterations in ventricular geometry influence myocardial wall stress and loading conditions [23]. These concepts provide a theoretical framework for interpreting geometric descriptors of ventricular remodeling and support the rationale for investigating isoperimetric and fractal properties in HCM.

This study included two groups of patients: 60 individuals with HCM and 60 controls without morphological and functional signs of LV hypertrophy. The baseline characteristics (Table 1) confirmed that the groups were well matched in terms of age and LV systolic function, ensuring a comparable clinical background. Sex distribution was balanced in the control group. In contrast, male sex was more prevalent in the HCM group, which is consistent with previously published registry data, potentially reflecting differences in disease presentation and diagnosis between sexes [24,25]. As expected, patients with HCM exhibited a distinct structural and tissue profile, characterized by increased MWT, mass, and markers of diffuse remodeling (T1 values), as well as focal fibrosis (LGE). Differences in volumetric parameters (EDVi and SV) were also observed, reflecting alterations in ventricular geometry and hemodynamics associated with the disease.

The phenotypic characteristics of the HCM group, as presented in Table 2, demonstrate a typical morphological spectrum of the disease, with a predominance of asymmetric hypertrophy in accordance with previously published data [1,26]. In addition, a substantial proportion of patients exhibited mitral valve and PM abnormalities and features of dynamic LVOTO, emphasizing the complexity of LV remodeling in HCM. These findings support the concept that HCM is not solely defined by myocardial thickening, but rather by complex alterations in ventricular geometry and intracavitary structures [4]. In this context, they provide a clear rationale for the development of a novel parameter designed to capture and quantitatively describe the geometric complexity of the LV derived from 2D CMR images.

Quantitative descriptors of ventricular geometry, including FD and isoperimetric indices (IPI and CC-IPI), were assessed in both investigated groups. A key finding was the absence of statistically significant sex-related differences in FD within the control group, in contrast to both isoperimetric indices (IPI and CC-IPI, Table 3), which demonstrated consistent and statistically significant differences between males and females. Moreover, conventional CMR parameters (Table 4) also demonstrated statistically significant sex-related differences, which is in line with well-established physiological differences in cardiac size and ventricular geometry between sexes [27]. The concordance between the sex-related differences observed in the isoperimetric indices and those observed in established CMR parameters suggests that these indices adequately reflect physiological variations in ventricular geometry. The absence of such differences in FD suggests that this parameter may be less sensitive to global variations in ventricular geometry, likely due to its contour-based nature, which primarily reflects local endocardial irregularity without accounting for the relationship between the contour and the enclosed cavity [16]. In contrast, isoperimetric indices integrate both contour length and cavity area, thereby capturing the relationship between the endocardial boundary and effective cavity size and providing a more comprehensive descriptor of ventricular geometry. From a theoretical perspective, this approach is grounded in the isoperimetric principle, which defines the circle as the optimal configuration in two dimensions, enclosing the maximal area for a given perimeter. Deviations from this configuration reflect increasing geometric irregularity, which can be quantified using perimeter–area-based indices [28,29].

When comparing patients with HCM and controls, both isoperimetric indices (IPI and CC-IPI) were found to be significantly higher in the HCM group (Table 5 and Figure 2A, all *p* < 0.001), further supporting their strong discriminative capacity. Similar differences were also observed for FD (Table 5, all *p* < 0.001). Importantly, these differences remained highly significant after stratification by gender, with consistently elevated values observed in both male and female subgroups (Table 5), indicating that the geometric alterations captured by these indices are independent of physiological sex-related variation. Nevertheless, although both FD and the isoperimetric indices were able to discriminate HCM patients from controls, the findings observed in the control group, together with the concordance of isoperimetric indices with established CMR parameters, led us to prioritize IPI and CC-IPI as more physiologically grounded descriptors of left ventricular geometry. Consequently, subsequent analyses and interpretation in the present study focused primarily on the isoperimetric model.

Building on these findings, a strong linear correlation between IPI and CC-IPI was demonstrated (R^2^ = 0.997, *p* < 0.001), reflecting a high level of agreement between the two formulations. The tightly clustered linear distribution observed in the scatter plot (Figure 2B) suggests that both indices capture closely related aspects of ventricular geometry. While CC-IPI theoretically provides a cavity-corrected measure by accounting for intracavitary structures, its calculation is more complex; thus, its potential implementation will be slightly more complicated than IPI. The strong agreement between these two parameters indicates that the “simpler” IPI retains all necessary and essential geometric information and can therefore be a more practical and reproducible metric for further routine application.

ROC curve analysis further demonstrated full discriminatory performance of isoperimetric indices for identifying HCM, with no overlap between HCM and control groups. IPI showed an AUC of 1.00, with an optimal cut-off value of 4.86, yielding both sensitivity and specificity of 100% (Table 6). Comparable performance was observed for CC-IPI, which also achieved an AUC of 1.00, with an optimal cut-off value of 5.01 and identical sensitivity and specificity (Table 6). Additionally, FD also showed strong discriminatory performance but a small overlap between the HCM and control groups was observed (Figure S1). Using a sensitivity-preserving threshold of FD ≥ 1.0808 (Table 6), FD identified all HCM patients (sensitivity 100%), while two control subjects were classified as HCM, yielding a specificity of 96.67%. Therefore, FD can be considered a complementary marker, while IPI and CC-IPI should be observed as the primary diagnostic indices due to their complete non-overlapping separation.

Beyond their discriminatory performance, correlations with conventional CMR parameters provide additional insight into the biological relevance of isoperimetric indices, as summarized in Table 7. These indices demonstrated strong associations with MWT and indexed LV mass, two key parameters routinely used for the diagnosis and assessment of disease severity and progression in HCM [1,4]. These findings indicate that ventricular geometric complexity increases in parallel with the severity of hypertrophic remodeling, suggesting that alterations in ventricular geometry are an integral component of the HCM phenotype [1,6,12,30].

In comparison, associations with volumetric parameters such as EDV, EDVi and SV were weaker, suggesting that geometric complexity is primarily driven by structural remodeling rather than chamber size alone. It should be noted that these findings are derived from 2D image analysis, and the lower correlation with inherently 3D volumetric parameters may partly reflect methodological differences between planar contour-based indices and volumetric measurements [4]. Notably, isoperimetric indices also demonstrated a moderate association with native T1 values, which reflect diffuse myocardial fibrosis and interstitial remodeling, indicating that global myocardial alterations contribute to changes in ventricular geometry [6]. In contrast, no association was observed with T2 values, which is consistent with the biological interpretation of T2 as a marker of myocardial edema rather than chronic structural remodeling [31].

It is important to note that no association was observed between isoperimetric indices and the extent of LGE. This likely reflects the different biological processes captured by these parameters, as LGE identifies focal replacement fibrosis, whereas geometric irregularity represents a global manifestation of ventricular remodeling [8,32]. The observed relationship with native T1 mapping, but not with LGE, is consistent with the concept that ventricular geometric complexity is more closely related to diffuse myocardial alterations than to focal fibrotic lesions, suggesting that isoperimetric indices reflect global myocardial and geometric remodeling [32].

Collectively, the present study represents, to the best of our knowledge, the first evaluation of the isoperimetric principle as a quantitative approach to assessing LV geometry in HCM patients. From a conceptual perspective, isoperimetric indices can be considered quantitative descriptors of ventricular shape efficiency. In physiological conditions, ventricular geometry tends toward configurations that optimize mechanical performance, whereas in HCM, this balance is disrupted, leading to increased geometric irregularity [10,33]. In this context, isoperimetric analysis provides a simple and interpretable metric linking ventricular form to functional and structural remodeling.

These observations suggest that the potential utility of geometric indices may extend beyond phenotypic characterization alone. Quantitative assessment of ventricular geometry may have applications in different clinical settings that warrant additional investigation. Although the present study was not designed to evaluate clinical outcomes or guide therapeutic decision-making, objective characterization of LV cavity morphology can provide additional information regarding the spatial distribution of remodeling in HCM. Thus, such geometric descriptors could potentially contribute to pre-procedural planning of septal reduction therapies by providing a more comprehensive assessment of ventricular architecture beyond conventional measures of wall thickness. Future studies are needed to determine whether isoperimetric indices provide incremental value in this setting.

## 5. Conclusions

Taken together, these findings support the concept that HCM represents not only a disease of myocardial hypertrophy but also a disorder of ventricular geometry. Isoperimetric indices provide a reproducible and physiologically grounded metric that captures global geometric remodeling and complements conventional CMR parameters. Importantly, IPI and CC-IPI should be interpreted as complementary to, rather than interchangeable with, FD-based analysis: while FD reflects scale-dependent endocardial contour complexity, isoperimetric indices quantify boundary length relative to cavity area and therefore describe a distinct aspect of ventricular geometric remodeling. Given their strong association with well-established markers of hypertrophy and their relationship with diffuse myocardial changes, these indices offer additional value in the phenotypic characterization of HCM. Additional studies with larger cohorts are warranted to further validate the potential role of IPI and CC-IPI, alone and in combination with FD and other geometric descriptors, in risk stratification and clinical decision-making.

## Figures and Tables

**Table 1:** Baseline characteristics of all enrolled subjects.

	HCM	Control	*p* Value
Age	42.95 ± 11.8	41.07 ± 11.15	0.371 ^a^
Gender (male)	44 (73.3%)	33 (55.0%)	0.036 ^b^
CMR parameters			
LV EF (%)	61.22 ± 6.06	60.18 ± 3.83	0.266 ^a^
MWT (mm)	20.31 ± 3.85	7.8 ± 1.61	<0.001 ^a^
EDV (mL)	175.85 ± 46.41	160.43 ± 26.41	0.028 ^a^
EDVi (mL/m^2^)	86.28 ± 18.2	79.31 ± 10.6	0.012 ^a^
SV (mL)	107.33 ± 29.05	96.25 ± 15.5	0.011 ^a^
LV mass i (g/m^2^)	91.32 ± 26.66	46 ± 7.37	<0.001 ^a^
T1 mapping * (ms)	1031.28 ± 50.71	974.81 ± 25.56	<0.001 ^a^
T2 mapping ** (ms)	53.33 ± 4.89	52.61 ± 3.49	0.388 ^a^
LGE *** (%)	11.2 ± 9.24	/	/

^a^ Independent samples *t*-test; ^b^ Pearson’s chi-square test. Abbreviations: HCM—hypertrophic cardiomyopathy; CMR—cardiac magnetic resonance; LV EF—left ventricular ejection fraction; MWT—maximal wall thickness; EDV—end-diastolic volume; EDVi—end-diastolic volume index; SV—stroke volume; LV mass i—left ventricular mass index; LGE—late gadolinium enhancement. *—number of subjects: 114; **—number of subjects 109; ***—number of subjects 60.

**Table 2:** Morphological and phenotypic characteristics of the HCM group.

CMR Parameters	N (%)
SAM	27 (45%)
LVOTO	21 (35%)
HCM type	
Asymmetric	53 (88.3%)
Apical	2 (3.3%)
Concentric	5 (8.3%)
Apical papillary muscle displacement	24 (40%)

Abbreviations: CMR—cardiac magnetic resonance; SAM—systolic anterior motion; LVOTO—left ventricular outflow tract obstruction; HCM—hypertrophic cardiomyopathy.

**Table 3:** Gender-based differences in isoperimetric indices and fractal dimension in the control group.

	Males	Females	*p* Value
FD	1.06 ± 0.01	1.06 ± 0.01	0.479 ^a^
IPI	4.34 ± 0.19	4.19 ± 0.18	0.003 ^a^
CC-IPI	4.49 ± 0.22	4.32 ± 0.18	0.002 ^a^

^a^ Independent samples *t*-test. Abbreviations: FD—fractal dimension; IPI—conventional isoperimetric index; CC-IPI—cavity-corrected isoperimetric index.

**Table 4:** Age and gender-based differences in CMR parameters in the control group (N = 60).

	Male (Mean ± SD)	Female (Mean ± SD)	*p* Value
Age	41.91 ± 10.64	40.04 ± 11.87	0.522 ^a^
CMR parameters			
LV EF (%)	59.30 ± 3.48	61.26 ± 4.02	0.048 ^a^
MWT (mm)	8.64 ± 1.50	6.78 ± 1.09	<0.001 ^a^
EDV (mL)	174.64 ± 22.92	143.07 ± 19.18	<0.001 ^a^
EDVi (mL/m^2^)	82.91 ± 10.18	74.90 ± 9.55	0.003 ^a^
SV (mL)	103.30 ± 13.16	87.63 ± 13.85	<0.001 ^a^
LV mass i (g/m^2^)	50.94 ± 5.06	39.96 ± 4.81	<0.001 ^a^
T1 mapping * (ms)	965.45 ± 19.76	985.68 ± 27.54	0.003 ^a^
T2 mapping ** (ms)	51.73 ± 3.48	53.61 ± 3.30	0.060 ^a^

^a^ Independent samples *t*-test. Abbreviations: LV EF—left ventricular ejection fraction; MWT—maximal wall thickness; EDV—end-diastolic volume; EDVi—end-diastolic volume index; ESVi—end-systolic volume index; SV—stroke volume; LV mass i—left ventricular mass index. *—number of subjects: 54; **—number of subjects 49.

**Table 5:** Comparison of isoperimetric indices and fractal dimension between HCM and control groups in the overall cohort and stratified by gender.

	HCM (Mean ± SD)	Control (Mean ± SD)	*p* Value
	Total		
IPI	6.33 ± 0.72	4.28 ± 0.2	<0.001 ^a^
CC-IPI	6.55 ± 0.75	4.41 ± 0.22	<0.001 ^a^
FD	1.13 ± 0.02	1.06 ± 0.01	<0.001 ^a^
	Males		
IPI	6.43 ± 0.69	4.34 ± 0.2	<0.001 ^a^
CC-IPI	6.65 ± 0.73	4.49 ± 0.22	<0.001 ^a^
FD	1.14 ± 0.02	1.06 ± 0.01	<0.001 ^a^
	Females		
IPI	6.05 ± 0.75	4.19 ± 0.18	<0.001 ^a^
CC-IPI	6.27 ± 0.75	4.32 ± 0.18	<0.001 ^a^
FD	1.12 ± 0.02	1.06 ± 0.01	<0.001 ^a^

^a^ Independent samples *t*-test. Abbreviations: HCM—hypertrophic cardiomyopathy; IPI—conventional isoperimetric index; CC-IPI—cavity-corrected isoperimetric index; FD—fractal dimension.

**Table 6:** Diagnostic performance of isoperimetric indices (IPI and CC-IPI) and fractal dimension based on ROC curve analysis.

Test Result Variable(s)	Cut-off ^a^	Sensitivity	Specificity
IPI	4.86	1.00	1.00
CC-IPI	5.01	1.00	1.00
FD	1.0808	1.00	0.97

^a^ The smallest cut-off value is the minimum observed test value minus 1, and the largest cut-off value is the maximum observed test value plus 1. All the other cut-off values are the averages of two consecutive ordered observed test values. For both IPI and CC-IPI, Youden’s index is 1.00, while for FD, Youden’s index is 0.97. Abbreviations: IPI—conventional isoperimetric index; CC-IPI—cavity-corrected isoperimetric index; FD—fractal dimension.

**Table 7:** Pearson correlation between isoperimetric indices and conventional CMR parameters for all subjects.

	IPI r (*p*)	CC-IPI r (*p*)
Age	0.134 (0.145)	0.127 (0.167)
LV EF (%)	0.064 (0.490)	0.062 (0.499)
MWT (mm)	0.867 (<0.001)	0.867 (<0.001)
EDV (mL)	0.223 (0.014)	0.217 (0.017)
EDVi (mL/m^2^)	0.256 (0.05)	0.253 (0.05)
SV (mL)	0.237 (0.009)	0.229 (0.012)
LV mass i (g/m^2^)	0.807 (<0.001)	0.803 (<0.001)
T1 mapping * (ms)	0.567 (<0.001)	0.567 (<0.001)
T2 mapping ** (ms)	0.120 (0.213)	0.118 (0.223)
LGE *** (%)	0.210 (0.107)	0.223 (0.087)

Abbreviations: IPI—conventional isoperimetric index; CC-IPI—cavity-corrected isoperimetric index; LV EF—left ventricular ejection fraction; MWT—maximal wall thickness; EDV—end-diastolic volume; EDVi—end-diastolic volume index; SV—stroke volume; LV mass i—left ventricular mass index; LGE—late gadolinium enhancement; *—number of subjects: 114; **—number of subjects 109; ***—number of subjects 60.

## Data Availability

The data that support the findings of this study are available from the corresponding author upon reasonable request and are not publicly available due to privacy and ethical restrictions.

## Supplementary Materials

The following supporting information can be downloaded at: https://www.mdpi.com/article/10.3390/diagnostics16142142/s1, Table S1: Slice-based variability of isoperimetric indices across study participants; Figure S1: Box plot illustrating the distribution of FD values in HCM patients and controls; Figure S2: Scatter plot demonstrating the relationship between IPI and FD (R^2^ = 0.9162); Figure S3: Scatter plot demonstrating the relationship between CC-IPI and FD (R^2^ = 0.9144).

## Abbreviations

The following abbreviations are used in this manuscript:

HCM	Hypertrophic cardiomyopathy
CMP	Cardiomyopathy
LV	Left ventricular
MWT	Maximum wall thickness
PM	Papillary muscles
SAM	Systolic anterior motion
LVOTO	Left ventricular outflow tract obstruction
CMR	Cardiac magnetic resonance
LGE	Late gadolinium enhancement
LVEF	Left ventricular ejection fraction
EACVI	European Association of Cardiovascular Imaging
FD	Fractal dimension
PACS	Picture Archiving and Communication System
SAX	Short-axis
BB	Black-blood
SV	Stroke volume
EDVi	Indexed end-diastolic volume
ESVi	Indexed end-systolic volume
ESC	European society of cardiology
L	Length
A	Area
IPI	Conventional isoperimetric index
CC-IPI	Cavity-corrected isoperimetric index
SD	Standard deviation
CV	Coefficient of variation
ROC	Receiver operating characteristic
AUC	Area under the curve
