# Supplementary material for: Left Ventricular Isoperimetric Properties in Hypertrophic Cardiomyopathy: A CMR-Based Analysis of Ventricular Geometry
Source: Diagnostics (Basel). 2026 Jul 8;16(14):2142. doi: 10.3390/diagnostics16142142 (PMC13407540; doi:10.3390/diagnostics16142142)
Supplement: Supplementary file 1 [file diagnostics-16-02142-s001.zip › diagnostics-4317309-supplementary.pdf]

Supplementary Material

Left Ventricular Isoperimetric Properties in Hypertrophic Cardiomyopathy: A CMR-Based Analysis of Ventricular Geometry

Maja Milošević Nale[1], Bojan Božić[2,*], Ivan Soldatović[3], Ljiljana Ranković-Ničić[1], Goran Lončar[1,3], Milica Milošević[1], Nikola Mitović[3], Vladimir Mihajlović[1], Ivana Petrović[1,4], Milovan Bojić[1,4], Milan Dobrić[1,3,*]

[1] Institute for Cardiovascular Diseases "Dedinje", Heroja Milana Tepića 1, Belgrade, Serbia; maja.a.milosevic@gmail.com (M.M.N); ljiljanabg80@yahoo.com (LJ.R.-N.); loncar_goran@yahoo.com (L.G.); milosevic.a.milica13@gmail.com (M.M.); vlada_sd@yahoo.com (V.M.); petrovicivana4@gmail.com (I.P.); dedinje@ikvbd.com (M.B.); iatros007@gmail.com (M.D.)
[2] University of Belgrade, Institute of Physiology and Biochemistry "Ivan Djaja", Faculty of Biology, Studentski trg 16, Belgrade, Serbia; bbozic@bio.bg.ac.rs (B.B.)
[3] Faculty of Medicine, University of Belgrade, Dr Subotića 8, Belgrade, Serbia; ivan.soldatovic@med.bg.ac.rs (I.S.); nikolamitovic@gmail.com (N.M.),
[4] Faculty of Medicine, University of Banja Luka, Save Mrkalja 14, Banja Luka, Bosnia and Herzegovina;
* Correspondence: bbozic@bio.bg.ac.rs (B.B.) and iatros007@gmail.com (M.D.)

Table S1: Slice-based variability of isoperimetric indices across study participants.

	IPI						CC-IPI					
	**Mean**	**SD**	**CV**	**N**	**Ng5**	**Ng5/N (%)**	**Mean**	**SD**	**CV**	**N**	**Ng5**	**Ng5/N (%)**
**Control**	4.28	0.51	11.90	11.18	1.28	11.52	4.41	0.53	11.93	11.18	1.95	17.33
**HCM**	6.33	1.41	21.95	11.53	8.67	75.39	6.55	1.45	21.76	11.53	9.27	80.68

Abbreviations: IPI - Conventional Isoperimetric Index; CC-IPI – Cavity-Corrected Isoperimetric Index, HCM - hypertrophic cardiomyopathy, SD – standard deviation, CV- coefficient of variation, N - total number of analyzed slices; Ng5 - number of slices with IPI / CC-IPI values exceeding 5; Ng5/N (%) - proportion of slices with IPI / CC-IPI values exceeding 5.

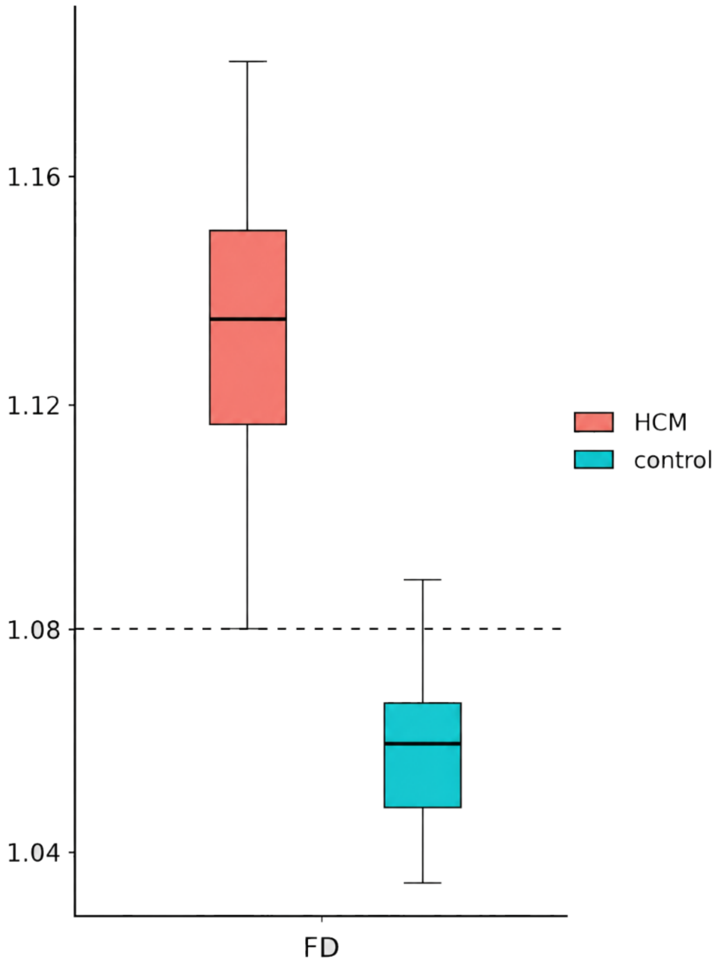

Figure S1. Box plot illustrating the distribution of FD values in HCM patients and controls. Abbreviations: FD – Fractal Dimension, HCM – hypertrophic cardiomyopathy.

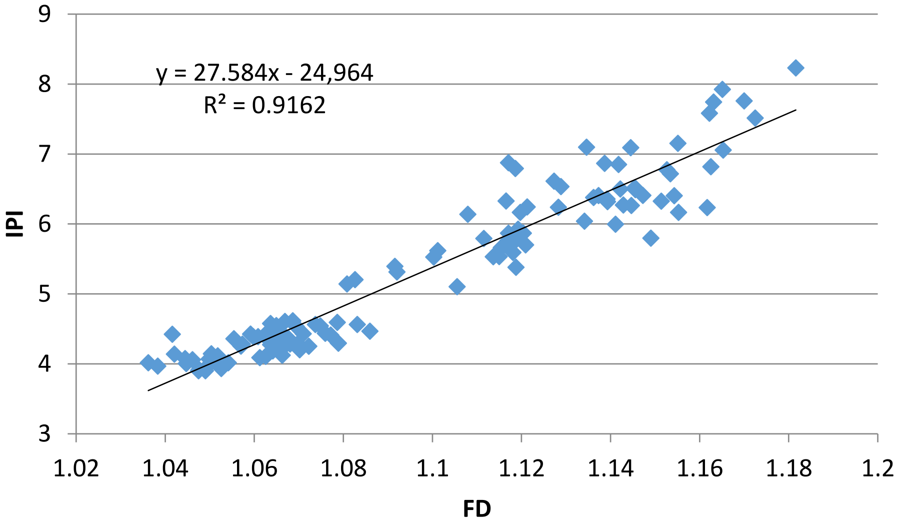

Figure S2. Scatter plot demonstrating the relationship between IPI and FD ($R^2 = 0.9162$). Abbreviations: IPI - Conventional Isoperimetric Index; FD – Fractal Dimension.

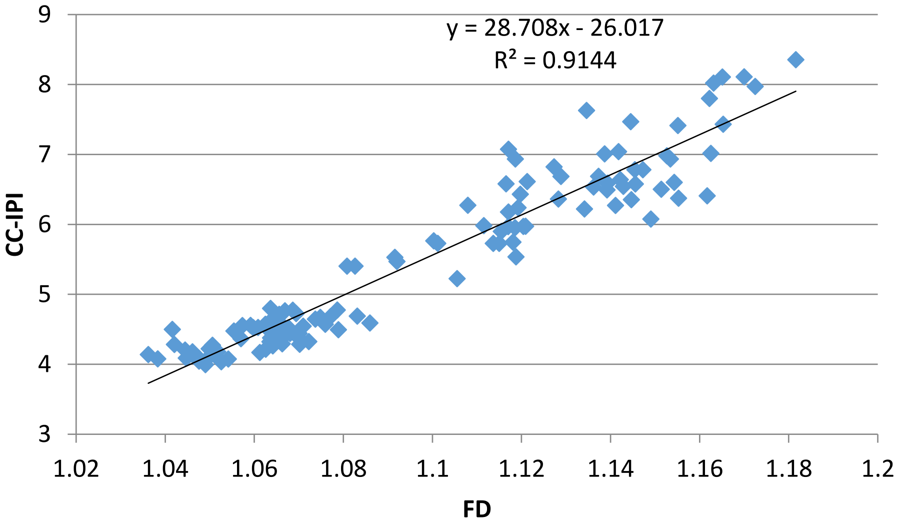

Figure S3. Scatter plot demonstrating the relationship between CC-IPI and FD ($R^2 = 0.9144$). Abbreviations: CC-IPI – Cavity-Corrected Isoperimetric Index; FD – Fractal Dimension.
